# Butane-1,4-di­ammonium hexa­fluoro­silicate

**DOI:** 10.1107/S1600536814001068

**Published:** 2014-01-18

**Authors:** Ali Ouasri, Ali Rhandour, Mohamed Saadi, Lahcen El Ammari

**Affiliations:** a Département de Physique-Chimie, Laboratoire de Chimie, Centre Régional des Métiers de l’Education et de la Formation, Souissi Rabat, Morocco; b Equipe de Physico-Chimie des Matériaux Inorganiques, Université Ibn Tofail, Faculté des Sciences, BP 133, 14000 Kénitra, Morocco; c Laboratoire de Chimie du Solide Appliquée, Faculté des Sciences, Université Mohammed V-Agdal, Avenue Ibn Battouta, BP 1014, Rabat, Morocco

## Abstract

The title compound, [NH_3_(CH_2_)_4_NH_3_]^2+^·SiF_6_^2−^, is a hybrid built from an organic butane-1,4-di­ammonium dication linked to a hexa­fluoro­silicate mineral anion. Both ions posses inversion symmetry. In the anion the Si atom is located on an inversion center, while in the cation the center of inversion is situated at the mid-point of the central —CH_2_—CH_2_— bond. The Si atom is surrounded by six F atoms, forming a slightly distorted SiF_6_^2−^ octa­hedron. These octa­hedra are linked to the organic cations through N—H⋯F hydrogen bonds, forming a three-dimensional network.

## Related literature   

For background to potential physical properties, see: Ouasri *et al.* (2003 ▶); Elyoubi *et al.* (2004 ▶). For similar compounds, see: Jeghnou *et al.* (2005 ▶); Rhandour *et al.* (2011 ▶); Ouasri *et al.* (2012 ▶, 2013*a* ▶,*b* ▶, 2014 ▶).
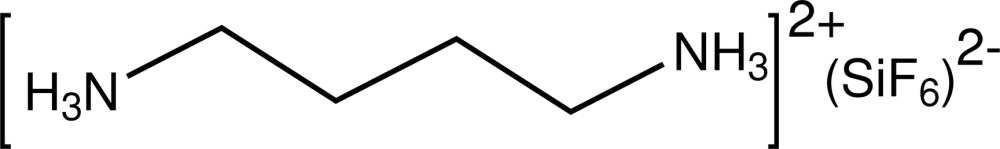


## Experimental   

### 

#### Crystal data  

C_4_H_14_N_2_^2+^·SiF_6_^2−^
*M* *_r_* = 232.26
Triclinic, 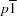

*a* = 5.796 (1) Å
*b* = 5.889 (1) Å
*c* = 7.774 (2) Å
α = 87.02 (1)°
β = 82.15 (1)°
γ = 61.87 (1)°
*V* = 231.79 (8) Å^3^
*Z* = 1
Mo *K*α radiation
μ = 0.31 mm^−1^
*T* = 296 K
0.36 × 0.32 × 0.27 mm

#### Data collection  

Bruker X8 APEX diffractometer
Absorption correction: multi-scan (*SADABS*; Bruker, 2009 ▶) *T*_min_ = 0.512, *T*_max_ = 0.640
5351 measured reflections
1285 independent reflections
1185 reflections with *I* > 2σ(*I*)
*R*_int_ = 0.025

#### Refinement  

*R*[*F* ^2^ > 2σ(*F* ^2^)] = 0.038
*wR*(*F* ^2^) = 0.108
*S* = 1.05
1285 reflections
61 parameters
H-atom parameters constrained
Δρ_max_ = 0.74 e Å^−3^
Δρ_min_ = −0.33 e Å^−3^

### 

Data collection: *APEX2* (Bruker, 2009 ▶); cell refinement: *SAINT-Plus* (Bruker, 2009 ▶); data reduction: *SAINT-Plus*; program(s) used to solve structure: *SHELXS97* (Sheldrick, 2008 ▶); program(s) used to refine structure: *SHELXL97* (Sheldrick, 2008 ▶); molecular graphics: *ORTEP-3 for Windows* (Farrugia, 2012 ▶); software used to prepare material for publication: *PLATON* (Spek, 2009 ▶) and *publCIF* (Westrip, 2010 ▶).

## Supplementary Material

Crystal structure: contains datablock(s) I. DOI: 10.1107/S1600536814001068/im2447sup1.cif

Structure factors: contains datablock(s) I. DOI: 10.1107/S1600536814001068/im2447Isup2.hkl

CCDC reference:

Additional supporting information:  crystallographic information; 3D view; checkCIF report

## Figures and Tables

**Table 1:** Hydrogen-bond geometry (Å, °)

*D*—H⋯*A*	*D*—H	H⋯*A*	*D*⋯*A*	*D*—H⋯*A*
N1—H1*NA*⋯F2	0.89	2.31	3.017 (2)	137
N1—H1*NA*⋯F3	0.89	2.39	3.134 (2)	142
N1—H1*NB*⋯F3^i^	0.89	2.02	2.890 (2)	167
N1—H1*NC*⋯F1^ii^	0.89	2.04	2.864 (2)	154

Symmetry codes: (i) 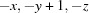; (ii) 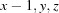.

**Fig. 1.**
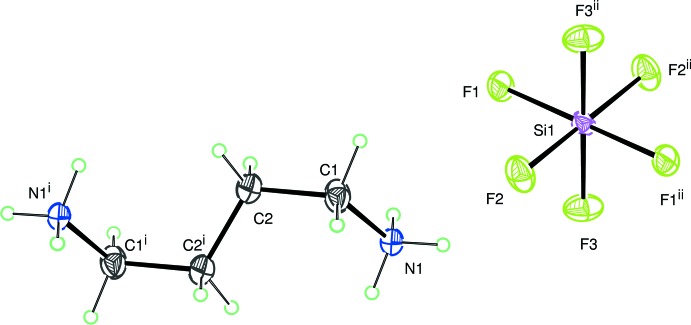
Molecular structure of the title compound with the atom-labelling scheme. Displacement ellipsoids are drawn at the 50% probability level. H atoms are represented as small circles.

**Fig. 2.**
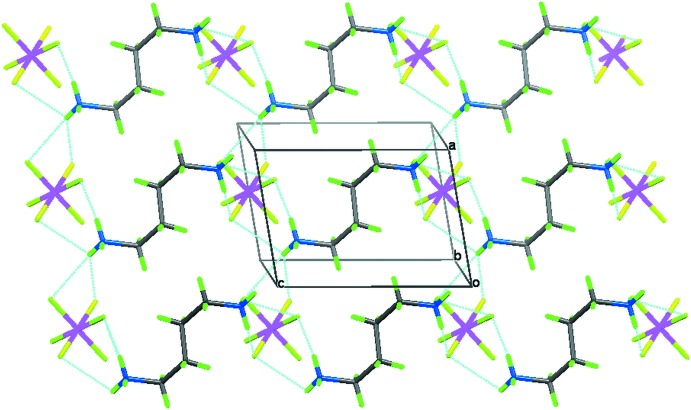
Three dimensional plot of the title compound, showing inorganic sheets linked through N–H···F hydrogen bonds to the organic layers (dashed lines).

## supplementary crystallographic information

### 1. Comment

The alkanediammonium halogenometallate salts family with the general formula (NH_3_(CH_2_)_n_NH_3_)*MX*_6_ where *M*: Sn, Si, Te and *X*: Cl, Br, I and F, have recently attracted the interest of many investigators due to their potential physical properties (Ouasri *et al.*, 2003; Elyoubi *et al.*, 2004). X-ray, thermal and vibrational studies of phase transitions have also been performed for highly related compounds belonging to the alkanediammonium halogenobismuthate salts family as the pentachlorobismuthate one (NH_3_(CH_2_)_n_NH_3_)BiCl_5_ (Jeghnou *et al.*, 2005; Ouasri *et al.*, 2012; Rhandour *et al.*, 2011; Ouasri *et al.*, 2013*a*; Ouasri *et al.*, 2013*b*; Ouasri *et al.*, 2014). The aim of the present paper was to study the recently synthesized butanediammonium hexafluorosilicate (NH_3_(CH_2_)_4_NH_3_)SiF_6_ crystal, by X-ray diffraction at room temperature.

The structure of the title compound is built up from inorganic anions linked to the organic cations trough hydrogen bonds. In this structure, all atoms are in general positions, except the silicon atom (Si1 (1/2, 0, 0) which is located at a crystallographic centre of inversion of the *P*1 space group. In addition, the centre of the bond C2—C2i is also situated on another crystallographic centre of inversion. The asymmetric unit therefore contains only one half of the organic cation and one SiF_3_ moiety. The remaining atoms of the unit cell are generated by symmetry operations.

The silicon atom is surrounded by six fluorine atoms building a slightly distorted SiF_6_^2-^ octahedron. SiF_6_^2-^ octahedra are linked to the organic cations through N–H···F hydrogen bonds producing an infinite two-dimensional layer parallel to (0 1 1) (Fig.2 and Table 1).

### 2. Experimental

(NH_3_(CH_2_)_4_NH_3_)SiF_6_ single crystals were obtained by slow evaporation, at room temperature, of an aqueous solution containing stoichiometric amounts of butane-1,4-diamine, NH_2_(CH_2_)_4_NH_2_, and H_2_SiF_6_ acid.

### 3. Refinement

H atoms were located in a difference map and treated as riding with C—H = 0.97 Å, and 0.893 Å for CH_2_ and N–H, respectively. Thermal parameters of all hydrogen atoms were refeined with *U*_iso_(H) = 1.2 *U*_eq_(methylene) and *U*_iso_(H) = 1.5 *U*_eq_(N) for the ammonium groups.

### Crystal data

**Table:**

C_4_H_14_N_2_^2+^·SiF_6_^2^^−^	*Z* = 1
*M**_r_* = 232.26	*F*(000) = 120
Triclinic, *p*1	*D*_x_ = 1.664 Mg m^−^^3^
Hall symbol: -p 1	Mo *K*α radiation, λ = 0.71073 Å
*a* = 5.796 (1) Å	Cell parameters from 1285 reflections
*b* = 5.889 (1) Å	θ = 3.9–29.6°
*c* = 7.774 (2) Å	µ = 0.31 mm^−^^1^
α = 87.02 (1)°	*T* = 296 K
β = 82.15 (1)°	Block, colourless
γ = 61.87 (1)°	0.36 × 0.32 × 0.27 mm
*V* = 231.79 (8) Å^3^	

### Data collection

**Table:**

Bruker X8 APEX diffractometer	1285 independent reflections
Radiation source: fine-focus sealed tube	1185 reflections with *I* > 2σ(*I*)
Graphite monochromator	*R*_int_ = 0.025
φ and ω scans	θ_max_ = 29.6°, θ_min_ = 3.9°
Absorption correction: multi-scan (*SADABS*; Bruker, 2009)	*h* = −7→8
*T*_min_ = 0.512, *T*_max_ = 0.640	*k* = −8→8
5351 measured reflections	*l* = −10→10

### Refinement

**Table:**

Refinement on *F*^2^	Primary atom site location: structure-invariant direct methods
Least-squares matrix: full	Secondary atom site location: difference Fourier map
*R*[*F*^2^ > 2σ(*F*^2^)] = 0.038	Hydrogen site location: inferred from neighbouring sites
*wR*(*F*^2^) = 0.108	H-atom parameters constrained
*S* = 1.05	*w* = 1/[σ^2^(*F*_o_^2^) + (0.0549*P*)^2^ + 0.127*P*] where *P* = (*F*_o_^2^ + 2*F*_c_^2^)/3
1285 reflections	(Δ/σ)_max_ < 0.001
61 parameters	Δρ_max_ = 0.74 e Å^−^^3^
0 restraints	Δρ_min_ = −0.33 e Å^−^^3^

### Special details

**Table:**

Geometry. All e.s.d.'s (except the e.s.d. in the dihedral angle between two l.s. planes) are estimated using the full covariance matrix. The cell e.s.d.'s are taken into account individually in the estimation of e.s.d.'s in distances, angles and torsion angles; correlations between e.s.d.'s in cell parameters are only used when they are defined by crystal symmetry. An approximate (isotropic) treatment of cell e.s.d.'s is used for estimating e.s.d.'s involving l.s. planes.
Refinement. Refinement of *F*^2^ against all reflections. The weighted *R*-factor *wR* and goodness of fit *S* are based on *F*^2^, conventional *R*-factors *R* are based on *F*, with *F* set to zero for negative *F*^2^. The threshold expression of *F*^2^ > 2σ(*F*^2^) is used only for calculating *R*-factors(gt) *etc*. and is not relevant to the choice of reflections for refinement. *R*-factors based on *F*^2^ are statistically about twice as large as those based on *F*, and *R*- factors based on all data will be even larger.

### Fractional atomic coordinates and isotropic or equivalent isotropic displacement parameters (Å^2^)

**Table:**

	*x*	*y*	*z*	*U*_iso_*/*U*_eq_	
C1	−0.1629 (3)	0.2744 (4)	0.3650 (2)	0.0394 (4)	
H1A	0.0138	0.2407	0.3801	0.047*	
H1B	−0.1739	0.1160	0.3876	0.047*	
C2	−0.3593 (4)	0.4783 (4)	0.4953 (2)	0.0435 (4)	
H2A	−0.3573	0.6396	0.4663	0.052*	
H2B	−0.3024	0.4297	0.6094	0.052*	
N1	−0.2086 (2)	0.3508 (2)	0.18267 (15)	0.0269 (3)	
H1NA	−0.0891	0.2252	0.1110	0.040*	
H1NB	−0.1946	0.4939	0.1603	0.040*	
H1NC	−0.3695	0.3792	0.1675	0.040*	
Si1	0.5000	0.0000	0.0000	0.02400 (16)	
F1	0.35813 (19)	0.26912 (18)	0.12542 (14)	0.0381 (3)	
F2	0.3234 (2)	−0.1206 (2)	0.12259 (14)	0.0426 (3)	
F3	0.2526 (2)	0.1424 (2)	−0.12459 (15)	0.0445 (3)	

### Atomic displacement parameters (Å^2^)

**Table:**

	*U*^11^	*U*^22^	*U*^33^	*U*^12^	*U*^13^	*U*^23^
C1	0.0304 (7)	0.0470 (9)	0.0266 (7)	−0.0069 (7)	−0.0029 (5)	0.0022 (6)
C2	0.0368 (9)	0.0649 (12)	0.0278 (7)	−0.0233 (8)	0.0014 (6)	−0.0109 (7)
N1	0.0262 (6)	0.0287 (6)	0.0236 (5)	−0.0117 (5)	0.0007 (4)	−0.0032 (4)
Si1	0.0193 (3)	0.0203 (3)	0.0312 (3)	−0.00873 (19)	0.00039 (18)	−0.00429 (18)
F1	0.0318 (5)	0.0310 (5)	0.0487 (6)	−0.0135 (4)	0.0061 (4)	−0.0182 (4)
F2	0.0412 (6)	0.0386 (5)	0.0505 (6)	−0.0251 (5)	0.0122 (4)	−0.0037 (4)
F3	0.0405 (6)	0.0350 (5)	0.0593 (7)	−0.0143 (4)	−0.0245 (5)	0.0034 (5)

### Geometric parameters (Å, º)

**Table:**

C1—N1	1.4853 (19)	N1—H1NB	0.8900
C1—C2	1.512 (2)	N1—H1NC	0.8900
C1—H1A	0.9700	Si1—F2^ii^	1.6757 (10)
C1—H1B	0.9700	Si1—F2	1.6757 (10)
C2—C2^i^	1.519 (4)	Si1—F1	1.6886 (9)
C2—H2A	0.9700	Si1—F1^ii^	1.6886 (9)
C2—H2B	0.9700	Si1—F3	1.6908 (10)
N1—H1NA	0.8900	Si1—F3^ii^	1.6908 (10)
			
N1—C1—C2	112.70 (13)	H1NB—N1—H1NC	109.5
N1—C1—H1A	109.1	F2^ii^—Si1—F2	180.0
C2—C1—H1A	109.1	F2^ii^—Si1—F1	89.09 (5)
N1—C1—H1B	109.1	F2—Si1—F1	90.91 (5)
C2—C1—H1B	109.1	F2^ii^—Si1—F1^ii^	90.91 (5)
H1A—C1—H1B	107.8	F2—Si1—F1^ii^	89.09 (5)
C1—C2—C2^i^	114.65 (19)	F1—Si1—F1^ii^	180.0
C1—C2—H2A	108.6	F2^ii^—Si1—F3	91.03 (6)
C2^i^—C2—H2A	108.6	F2—Si1—F3	88.98 (6)
C1—C2—H2B	108.6	F1—Si1—F3	89.04 (6)
C2^i^—C2—H2B	108.6	F1^ii^—Si1—F3	90.96 (6)
H2A—C2—H2B	107.6	F2^ii^—Si1—F3^ii^	88.97 (6)
C1—N1—H1NA	109.5	F2—Si1—F3^ii^	91.02 (6)
C1—N1—H1NB	109.5	F1—Si1—F3^ii^	90.96 (6)
H1NA—N1—H1NB	109.5	F1^ii^—Si1—F3^ii^	89.04 (6)
C1—N1—H1NC	109.5	F3—Si1—F3^ii^	180.0
H1NA—N1—H1NC	109.5		

Symmetry codes: (i) −*x*−1, −*y*+1, −*z*+1; (ii) −*x*+1, −*y*, −*z*.

### Hydrogen-bond geometry (Å, º)

**Table:**

*D*—H···*A*	*D*—H	H···*A*	*D*···*A*	*D*—H···*A*
N1—H1*NA*···F2	0.89	2.31	3.017 (2)	137
N1—H1*NA*···F3	0.89	2.39	3.134 (2)	142
N1—H1*NB*···F3^iii^	0.89	2.02	2.890 (2)	167
N1—H1*NC*···F1^iv^	0.89	2.04	2.864 (2)	154

Symmetry codes: (iii) −*x*, −*y*+1, −*z*; (iv) *x*−1, *y*, *z*.
